# “In Space” or “As Space”?: A New Model 

**DOI:** 10.3390/life2030243

**Published:** 2012-08-31

**Authors:** Charles H. Smith, Megan Derr

**Affiliations:** 1 Center for Biodiversity Studies, 1906 College Heights Blvd. #11080, Western Kentucky University, Bowling Green, KY 42101, USA; 2 Emergint Technologies, 455 South 4^th^ Street, Suite 1250, Louisville, KY 40202, USA; E-Mail: megan.derr@emergint.com

**Keywords:** entropy maximization, stream systems, complex systems, living systems, spatial extension, space

## Abstract

In this analysis natural systems are posed to subsystemize in a manner facilitating both structured information/energy sharing and an entropy maximization process projecting a three-dimensional, spatial, outcome. Numerical simulations were first carried out to determine whether *n* × *n* input-output matrices could, once entropy-maximized, project a three-dimensional Euclidean metric. Only 4 × 4 matrices could; a small proportion passed the test. Larger proportions passed when grouped random patterns on and within two- and three-dimensional forms were tested. Topographical patterns within 31 stream basin systems in the state of Kentucky, USA, were then similarly investigated, anticipating that the spatial configuration of elevations within each basin would provide evidence of evolutionary control when interpreted as internal group relations. Twenty-eight of thirty-one of the systems pass the test unambiguously, with the remaining three approaching or reaching passage when sampling density is increased. Two measures of subsystem-level redundancies are also introduced; these show: (1) surprisingly, minimized internal redundancy levels at the four subsystems level of analysis of the stream systems (as opposed to the five or six, in contrast with the simulations), and (2) much lower average levels than those obtained in the simulations at the same dimensions, both suggesting a preferred evolutionary path under real world conditions.

## 1. Introduction

Historically, treatments of the three-dimensionality of mesoscale nature have remained largely on a philosophical/mathematical plane [1,2,3,4]. In perhaps the most famous logical positivist effort to establish a formal version of empiricism [5], the scheme, based on a phenomenalistic approach to absolute location, was defeated by an inability to provide a logical definition for the connective term “located at” [6]. This should give some pause for thought; perhaps the notion that the events going on around us happen “in space” should be replaced with a different concept: that they happen “as space.” The Empiricist tradition we have developed to such a great extent is not geared to this type of thinking, however. As a result, most science features reductionism (with its emphasis on the study of efficient causes), an approach not well-suited to dealing with bigger questions, especially those characterizing the operation of complex systems.

Suppose instead that space has properties implicit in each individual thing that makes it up. Space itself could be the result of some organizational principle whose results are emergent at all levels of the scale hierarchy. The principle could be probabilistic in its enaction, yet, by virtue of its nature, confining in such a way as to make many kinds of results impossible *a priori*.

This kind of thinking might be taken in many directions; one is toward the doctrine of final causes. This, one of the four classical Aristotelian causes, is most familiar in its application to design—for example, to the process of realization of artistic intent, as when a sculptor plans out his work before actually setting upon it. It is also relatable to teleology, and usually is. But there is no reason why teleology—especially as dictated by first causes (*i.e.*, supranatural intervention)—must be involved. In fact, good examples of final causation may be observed in nature that need not be related to miraculous intervention or intelligent design in general. One of the best is the DNA molecule.

Here is a structure whose evolution has taken place not only at its own level of organization, but simultaneously in response to a world of events beyond it. It can hardly be understood otherwise. The purpose of DNA is to build, from scratch as it were, a supra-DNA entity that fits into a pre-existing supra-individual environment. It is in essence an evolving program, one that is observing a final cause: the creation and maintenance of a living organism whose existence contributes to the operation of the ecological space supporting it.

In the spirit of exploration many years ago the senior author began to consider what kind of organizational principle might act as a wholly “natural” final cause. I soon came across writings of Benedict de Spinoza that could be related to modern ideas on subsystemization. A paper followed [7], developing a kind of “lazy universe” model based on combinatorial properties of branching hierarchical structures. This provided a model of historical relations among subsystems (as might be applied, for example, to the sequential evolution of tectonic plates, or the ontological development of organal systems in the body), but not a model of spatial extension *per se*; that is, an ecological understanding of natural function. Several years later, again using Spinozian thinking as my guide, I came up with such a model.

This is not the place to attempt to go through this model in detail [8], but very briefly it can be described as follows. It is proposed that all natural structures are internally organized on the basis of the interrelations—energy and information exchange—among some small number of subsystems that in sum make them up. Subsystemization serves the dual function of channeling information flow to (temporarily) maintain structure, while creating an entropy maximization process that ultimately yields (or *is*) spatial extension. Spatial extension is thus viewed in terms of the conditions of subsystemization, which are universal in all systems. That does not necessarily mean that all systems, at all scales, will exhibit obvious signs of such subsystemization, which may be more about energy and information flow than about structure *per se*. Still, rather large scale systems such as stream basins and the interior zones of the earth might exhibit interpretable patterns, as might some biological systems (e.g., butterfly wing color patterns, or spatial variation in brain function as exhibited by magnitudes of blood flow or electrical activity). For years the senior author has been working through related simulations and real world system pilot studies, all of which have produced successful results, but it was only recently that a good enough data set became available to report on. The approach we have followed here starts with simulations, and proceeds with what might be considered natural experiments.

The real world system investigated has been chosen for its relation to the authors’ actual backgrounds, because it provides a clear example of the approach, and because data sets on other potentially exploitable subjects could not be obtained. It is not a biological example (though one could argue it is an ecological one, as topography is the net result of biogeochemical processes), but biological applications are not difficult to imagine, as will be discussed later.

## 2. Simulations and Analysis

### 2.1. Introduction and Simulations

Geographers have been making use of entropy maximization analysis techniques for over four decades [9,10]. Typically, the immediate goal is standardization of an ij/ji place-to-place flows matrix (whether of commodities, people, or some kind of information) such that absolute size/magnitude trends are removed; residual patterns are then examined for possible interpretations related to the relative remoteness of the places of origin involved. In almost all cases, the flows monitored are unaggregated; that is, no initial structure is assumed, and the data consist of an array of inputs-outputs—for example, forty towns and cities in a particular area which are all individually interacting with one another. In the study here, however, location-connected data were first grouped into class structures whose measured spatial interrelations were then entropy-maximized to investigate whether the resulting matrices bore evidence of a particular kind of spatial causality. Specifically, we examine the proposition that spatial extension itself might represent a standing projection of information exchange operationalized as a universal form of subsystemization within (all) natural systems. To our knowledge this is a completely novel approach. The first step in the analysis consisted of simulations designed to determine whether matrix configurations exist that could even in theory mimic the kind of dimensional projections envisioned.

### 2.2. Simulations

In the simulations, numerous matrices of varying dimension (3 × 3, 4 × 4, 5 × 5, and 6 × 6) filled with random numbers (simulating the universe of theoretically possible information flow/similarities data) were entropy-maximized, then examined for whether any of the resulting configurations corresponded to a Euclidean space. This can be determined through metric multidimensional scaling. “Successful” configurations not only had to correspond to an unambiguous three-dimensional geometric representation—as any zero-stress solution in three dimensions would—but also consist of element scores that as a group were equally weighted on the three cardinal orthogonal axes (that is, each point in the final spatial projection would be both the same distance from the origin, and the same set of distances from one another).

**Figure 1:**
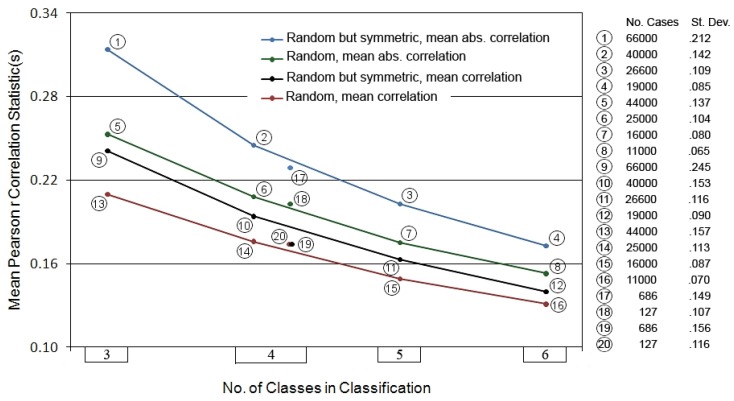
Summary of spatial projection simulations for matrix configurations of dimension 3 × 3 through 6 × 6. Circled numbers refer to data at the right margin giving the number of simulations in each test and the standard deviations accompanying the mean values plotted. The plotted numbers are the two *mmr* and two *mamr* values obtained at each dimensionality (= “no. of classes in classification”). Colored line coding connecting points is for readability purposes only. Point values 17 through 20 are compiled from subsets of the data leading to point values 2, 6, 10, and 14, respectively. See text for further explanation.

Thus the simulations examined a random sample of the universe of all possible combinations of *n* × *n* metric intra-relations, anticipating that some percentage of these—corresponding to real, or possible real, world conditions—would meet the posed conditions. Two sets of random numbers tests were run for each dimensionality: one in which the *n* × *n* matrices were filled with randomly-generated six-digit numbers, and one in which they were filled with the random numbers, but in an ij = ji symmetric pattern. In total nearly five hundred thousand sets of data were so treated (see the right margin of Figure 1 for the totals at each dimensionality). The entropy maximization operation applied was double-standardization (*i.e.*, bistochastization [11]), a technique in which all rows, and then columns, and then rows (and so on), are alternately standardized to *z *scores until the resulting matrix elements converge to stable values [12,13,14,15]. About a dozen different forms of *z* score arrays result (e.g., *n* × *n* different scores, *n* different scores symmetrically arranged, *n* different scores asymmetrically arranged, *etc*.), but all but one of these classes of patterns—a symmetric matrix of scores with the highest values in the i = j diagonal—will not secondarily project the spatial metric described above.

Only matrices of dimensionality *n* = 4 did produce such results. 127 of 25,000 (.51%) of the fully random 4 × 4 matrices double-standardized to the symmetric pattern, while 686 of 40,000 (1.72%) of the symmetric random 4 × 4 matrices did. These are small proportions, but a hopeful outcome: at the very least, they provide a firm starting point against which tests on real world systems might proceed. Two statistics describing the mean degree of structural redundancy within these systems were also compiled from the correlation matrix derived from each initial (random numbers) matrix: (1) the overall mean Pearson correlation coefficients making up each matrix (“*mr*”); (2) the mean of the absolute values of the column means of the *n* vectors of *r* values (*i.e.*, the mean of the deviations from zero of the *n* vector means of each matrix, “*amr*”). In either instance the lowest possible mean *r* value is zero, though invariably by chance there will be some net levels of inter-correlation. The first statistic thus captures net degree of inter-subsystem redundancy, whereas the second accumulates any vector mean differences away from zero, whether negative or positive.

In the simulation analyses to be described, the results across thousands of initial matrices were next averaged to produce the final summary statistics (means) for each set of results (1) and (2) above; these are hereafter referred to as *mmr* and *mamr*, respectively. Figure 1 summarizes the results of the simulations.

For each matrix dimensionality of three through six in Figure 1 there are four points plotting the summary *mmr* and *mamr* values for each set of simulations (the two redundancy measures combined with the fully random and symmetric random conditions). In addition, values 17 through 20 represent the plotted means for the particular matrices that passed the symmetric *z* scores test (at dimension 4 × 4). Two trends are clearly apparent across all the results: the *mmr* and *mamr* values trend uniformly downward as dimensionality increases, as do the associated standard deviations. Note again that these are “unconstrained” results; that is, the random numbers entered in for analysis have no prior spatial connection.

Given the small but non-negligible portion of the simulations that do pass the spatial projection test (and their varying levels of internal redundancy), it becomes more reasonable to consider whether system patterns in a real world—or first, perhaps, simulated real world—context might do the same (as it is apparent that persistent flows of material/information in real space are often manifest as standing structural patterns). Some years ago, the senior author carried out additional simulations involving two- or three-dimensional spaces populated by randomly-grouped point patterns. These were executed in the same fashion as described above, but in these instances the initial matrix elements consisted of spatial autocorrelation statistics (*i.e.*, assessing the varying grouped mean nearnesses of all pairings of sets of points in a particular simulation). For several series of simulated two-dimensional systems, about five percent of the patterns produced (that is, when grid-sampled locations were randomly grouped into *n* = 4 classes) passed the double standardization-based spatial projection test (the associated *mmr* values varied widely, from about 0.05 to 0.20). Other simulations followed on some simple three-dimensional systems, including simulated latitudinal zones around spheres, and concentric zones within spheres. These systems produced higher rates of passage of the spatial projection test, up to fifty percent in the case of the concentric zonations [16]. These too are encouraging results, as we might expect the rate of evolution to increase as milieus encouraging such change increase over time.

It is fair to ask what the *mr* and *amr* statistics might “look like” under real world conditions. In simple systems such as molecules this is difficult to say at this point, but in more complicated systems exhibiting complex textures and patterns, some guesses might be made. For example, one might expect higher *mr* and *amr* values to be associated with systems in early stages of development, as in biological ontogeny, or a newly evolving stream basin system containing lakes, swamps, and waterfalls (and thus a deficiency of gentle, regular, slopes). Lower means might be associated with mature stable systems; higher ones again, for disturbed systems such as newly pirated stream basins, or brains exhibiting the effects of Alzheimer’s disease. Note, however, that there is no one visible “pattern” to look for, since equally high levels of spatial nonrandomness can be expressed through a variety of configurations ranging from regular grid patterns to highly clustered ones [17]. That said, spatial autocorrelation measures can be used to identify nonrandomness of pattern, whatever the details of the form of the pattern might be.

### 2.3. Kentucky Stream Basins Analysis

The senior author has performed pilot analyses on the patterns exhibited by several kinds of real world systems in an effort to determine whether they too pass the spatial projection test. The early results seem to suggest so, but most recently a more rigorous study on a set of stream basins in Kentucky was undertaken. Thirty-one basins were selected; to avoid possible complications these were chosen for their relative uniformity of size (ranging in area from 2.8 to 6.6 square miles), outflow into streams of markedly larger size, and absence of complicating surface conditions such as strip mining and karst topography. Basin limits were established manually from USGS topographic maps; elevations across each basin were then grid-sampled using ArcGIS platform data. Three sampling densities were applied for comparative purposes: for the 1× sampling the real-world distance from each sample point on the surface to each of its six nearest neighbors was 860 feet, for the 4× sampling, 430 feet, and for the 16× sampling, 215 feet.

It is a given that every point on the terrestrial surface of the earth constitutes a physical outcome devolving from co-mingling forces of elevation and reduction. These outcomes produce a range of gravitational potential energies that may be expected to interact with one another, especially with respect to their baseline, as conditions evolve. In the context of the present study, it was reasoned that these forces might internally organize, *de facto*, through a subsystemization manifest in functional zones of elevation—that is, into subzones whose definition is *not* merely statistical, but actually structurally responsive to one another in an information-sharing, surface reducing/adjusting sense.

How this happens at the microscale is bound to be tied to all sorts of processes invoking both larger- and smaller-scale systems responding to a myriad of influences. Adrian Bejan’s constructal theory [18] addresses this by suggesting that natural systems evolve in such a manner as to better facilitate the currents of vital materials that flow through, and nourish, them. At different scales, the flows involved may be of entirely different makeups—people in urban commuter systems and blood in the human vascular system, for example—but according to Bejan’s theory, all systems exhibit a tendency to continue to structurally facilitate the flow. A similar understanding is invoked here, but the suggestion is made that the feedbacks that have evolved to operationalize such change flow primarily at the level of subsystem interactions. So, and for example, a stream basin disrupted by a sudden piracy or baseline change event might respond most efficiently through shifts in topographical configuration mediated by an entropy maximization process; that is, through feedbacks passed at the subsystem level (in this case, ranges of potential energies expressed as functional zones of elevation). In Bejan’s theory flows self-organize at different scales and rates dependent only on the ability of hierarchies of such to continue the process; in the model entertained here this is also true, though emergent structure is possible only when entropy is maximized among the subsystems in such a fashion as to correspond to an extended space metric.

**Figure 2:**
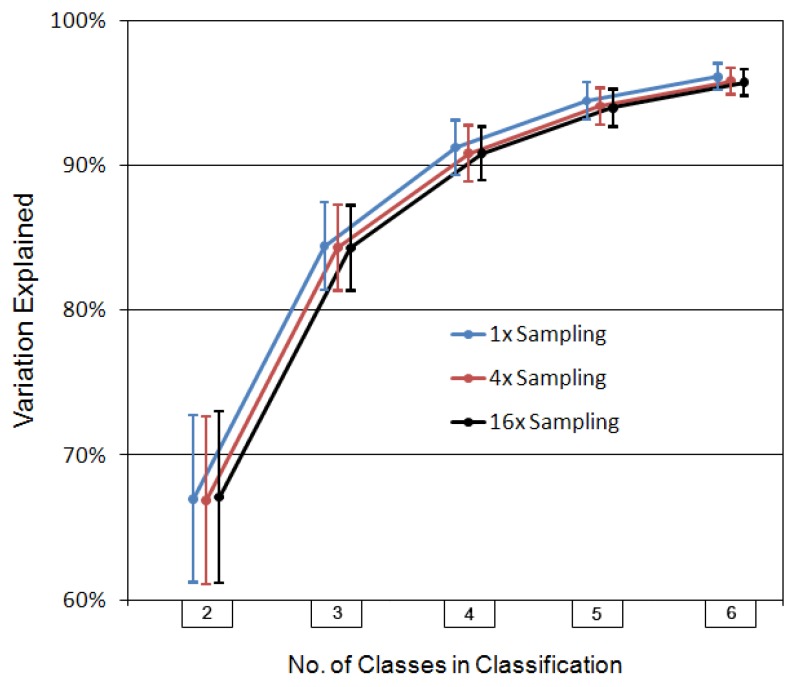
Summary of variation explained statistics obtained for the clustering of the stream basins data (vectors) into two through six classes. The plotted points are the mean (n = 31) variations-explained for each classification, at three fineness levels of sampling. Colored line coding connecting points is for readability purposes only.

The earlier pilot studies suggest that within some natural systems, the “imposed current” (in Bejan’s terminology) is so consistent and/or stable that physically observable boundaries between the subsystems emerge and persist. In more varying and/or multi-causal systems, however, the underlying organization may only be evident through the pattern of secondary indicators of its operation such as temperature or pressure. In the present instance, the topography itself reflects the impact of systemic interactions of gravitational forces. The central matter of interest here is whether evidence can be found that the system is self-organizing as a function of group relations, instead of a myriad of independently operating singularities. For each basin regular (triangular grid) samples of elevations were first classified into three, four, five, and six class structures using a nonhierarchical, information statistic-based, clustering algorithm [19]. Figure 2 summarizes the results of the clustering operations.

The summary statistics displayed in Figure 2 are very consistent, even considering they are based on only thirty-one cases. Variation explained increases rather smoothly, at least on the average, with number of classes. This was not unexpected, as only very unusual data will produce models in which a higher number of classes end up explaining less of the variation than a lower number.

**Figure 3:**
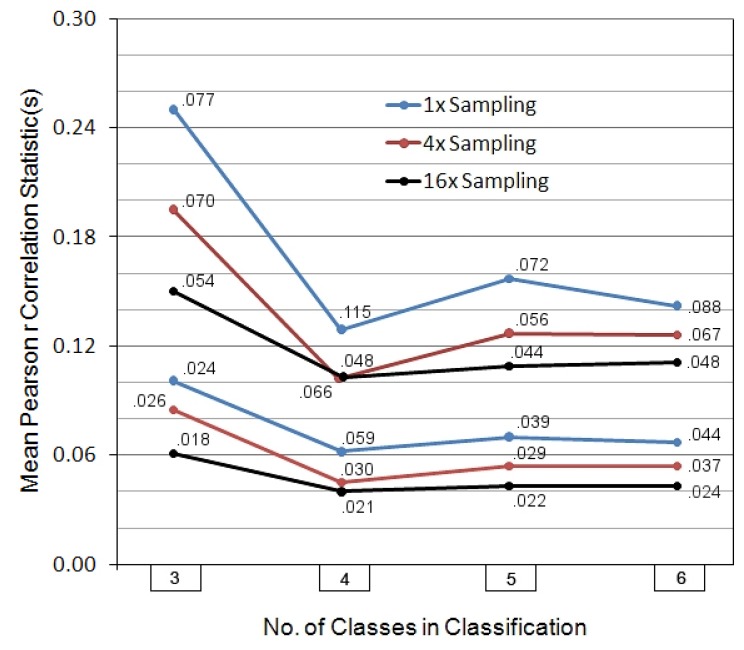
Summary of spatial subsystemization properties of topography in 31 Kentucky stream basins, based on three fineness levels of sampling. The plotted values in the top three sets of four points are the *mamr* values; those in the bottom three are the *mmr* values. The associated standard deviations are written out next to each plotted value. Colored line coding connecting points is for readability purposes only. The results as displayed here are in marked contrast to the simulation results shown in Figure 1, suggesting that the spatial expression of the basins is related to a functional (not just statistically described) subsystemization process.

A simple sum-of-squares-based (*i.e.*, not contiguities-based) spatial autocorrelation algorithm [20] was then applied to the inter-class patterns; the non-proportionalized summary spatial autocorrelation statistics thus generated became the elements of each initial data matrix (as was the case with the two- and three-dimensional simulations mentioned earlier). These statistics were then double-standardized. The results of this analysis are shown in Figure 3. 

Figure 3 displays the full set of *mmr* and *mamr* statistics across the thirty-one streams, relaying the results at the three sampling densities. All six patterns of results are roughly similar across the four matrix dimensionalities, though as one would expect, with higher fineness of sampling the details of pattern are better exposed, and all of the *mmr* and *mamr* values and their standard deviations drop. More importantly, these values are considerably lower than those evidenced in the simulations data in Figure 1, and it is apparent that once actual spatial relations are taken into account there is no longer a smooth pattern of decrease in means as the number of classes increases. In fact, the simulations notwithstanding, *mmr* and *mamr* actually minimize—absolutely—at the four-class level, not at the five- or six-. This is difficult to explain if there is not an actual, significant, structural causality operating at that level of subsystem definition. One of the earlier-mentioned pilot studies, though more crudely organized, came up with similar (though not quite so conclusive) results on another twenty-five basins of more widely varying size and geographical location, the *mmr* values lowering only very little, on the average, from the four- to five- and six-class solutions (four-class, 40% lower than three-; four- and five- very nearly the same; six-, only 13% lower than four-) [21].

For the four-class solutions, 28 of the 31 16× spatial autocorrelation matrices do double-standardize to a symmetric state passing the spatial projection test described earlier. 28 of 31 also pass at the 4× sampling density, but only 18 of 31 at the 1× (again, likely reflecting the poorer resolution connected to the coarser samples). Parallel analyses employing two other metric spatial autocorrelation measures produced similar results. Remembering that the two-dimensional simulations only yielded about five percent success rates, this is remarkable. In theory, however, all 31 should have passed, so as a check the three nonconforming basins were subjected to a special 64× sampling (capturing even more detail in the topography), whereupon two of the three then produced symmetric *z* scores, while in the third the mean ij / ji *z* scores difference (0.1306) had been reduced by about forty-four percent, suggesting that yet higher sampling densities might eventually complete the convergence; that is, to a mean ij / ji difference of 0. (In the earlier pilot study, 24 of 25 passed, with the twenty-fifth, an outlier in terms of basin size and some other characteristics, narrowly missing.)

It possibly will be objected that the main tests merely represent a reconstruction of three-dimensionality that was already built into the setting, but this seems not to be so. First, the *mmr* and *mamr* statistics (for 16×, *n* = 4, 0.0398 and 0.1029, respectively) for the case study are much lower than those connected with any of the parallel simulations (range: 0.1739 to 0.2287): they are thus a subpopulation which is in some sense special. Second, a test of the “reconstruction” hypothesis was fashioned by reclassifying each vector of elevations into two arbitrary sets of four classes markedly differing from the actual classifications in the case study (the highest or lowest 60% of the point elevations were summarily grouped into one class, with the remaining points grouped in elevation order into three smaller, equal-sized, classes), and for both 1× and 16× vectors. After the associated spatial autocorrelation statistics were calculated and subjected to double standardization, only 18 of 62 of the 1× systems passed the test, and 2 of 62 of the 16× systems. The *mmr* and *mamr* statistics derived from these last analyses (for 1×, 0.1792 and 0.3398; for 16×, 0.1522 and 0.2768, respectively) approached or exceeded those for the original simulations on random systems; that is, they were very much higher than those from the case study analyses [22].

## 3. Discussion

As long as physical expression in three dimensions is viewed as an *a priori* property of spatial extension—as opposed to one which emerges on a system-by-system basis—its reason for being will attract no great amount of interest. On the other hand, if dimensional projection is determined to be a property residing primarily *within* individual systems, important rewards might emerge from this fact. As mentioned earlier, the present work extends studies published in the 1980s [7,23] based on concepts that originated over three hundred years ago with the philosopher Benedict de Spinoza. Nevertheless, both the simulations and empiricism resulting are fairly conventional and straightforward, and should be relatively easy (uncostly) to further test or extend. There are good reasons for looking further into this matter. Briefly, if (all) other systems follow this pattern of organization—as its Spinozian basis argues—there might be many instances where such second-order analyses could usefully expose subtle conditions of, and trends in, their performance/operation [24]. In the stream basin systems described here (as well as in the nearly as many again examined in the earlier pilot study) a considerable range of levels of internal structural redundancy is evident, even when the systems pass the spatial projection test. This is what we should expect from naturally evolving systems, in which early growth stages are dominated by positive feedbacks and rapid change, and mature states are more typified by redundancy-reducing negative feedback processes that enhance diversity and stability. This is well known to happen in ecological systems, where community succession from barren beginnings usually starts with fast-growing, opportunist forms, but eventually favors slower-growing but longer-lasting species [25]. As the system changes over from a focus on competitive individual growth to the development of longer-living, cycle-perpetuating forms, flow access (of materials/information) becomes more extensive, allowing the development of more complex negative feedback paths. It is an important characteristic of the present model that it recognizes both the fact of varying internal system disequilibrium levels, and a means for their (static) descriptive measure [24]. In one of the pilot studies [26], the spatial autocorrelation properties of the four main internal zones of the earth (core, outer core, mantle, and surface zones) were investigated by the senior author using methods similar to those described here, and for the current time period, ninety-five million years ago, and two hundred million years ago (the analyses were based on three-dimensional grid-based samples of about sixteen thousand points each). The geostructural relationships among the four primary zones are of such long standing (extending back billions of years) that one might expect their limits to have very nearly attained dynamic equilibrium with one another, and in fact the primary mean correlation values (*mr*) produced in each instance ranged from about 0.0010 to 0.0020–values [27] considerably closer to zero than any of the simulations discussed earlier that passed the spatial projection test (and also lower than almost all of the nearly five hundred thousand matrices tested overall) [28]. Bejan would probably view these results as indicative of a system with a highly worked-out hierarchical “design,” that is, one which over a long period of time has developed such that the “imposed currents” which flow through it nourish a great complexity of structure. We would agree with this, but suggest that an explicitly spatial feedback element that would complete the causal picture is lacking in the Bejan model.

The model discussed here considers the nature of spatial extension itself, through the patterns that secondarily characterize it. If it can be proved valid, it will apply equally to all existing natural systems’ internal characteristics, and how these change over time. Concerning stream basins in particular, a process of ongoing internal adjustments can be imagined wherein the feedbacks required to maintain the overall system’s integrity are ordered *de facto* into an effective matrix of discrete information flows whose balance most likely operates within a limited range of mathematically-interpretable solutions. This could lead ultimately to a generalizable understanding of the interplay among the forces of landscape surface emergence, subsidence, deposition, and erosion.

Ultimately, however, the most important tests—and hopefully applications—of this model will fall within the biological sciences. Among the pilot studies mentioned earlier were analyses performed on color patterns on butterfly wings [29] and the internal structure of amino acids [30]. Both produced results consistent with the present model, if based on rudimentary data. More provocative results might be obtained by workers in these fields, using improved measurement concepts and techniques, and more extensive data. The most interesting tests of all, however, could come from medical diagnostic imaging data. Various global measures of brain function in particular—characteristics such as temperature, electrical activity or blood flow—seem custom made for the kind of pattern and internal disequilibrium analyses applied here to the topographical systems. Successful models might make it possible to predict physiological pathologies long before onset of overt symptoms, facilitating preventive treatments. At present we depend on direct observation/monitoring to identify degenerative conditions, but second-order pattern analyses of the type described here might ultimately identify more subtle, and/or earlier, warning signs.
